# Native T1 Mapping-Based Radiomics for Noninvasive Prediction of the Therapeutic Effect of Pulmonary Arterial Hypertension

**DOI:** 10.3390/diagnostics12102492

**Published:** 2022-10-14

**Authors:** Yue Wang, Lu Lin, Xiao Li, Jian Cao, Jian Wang, Zhi-Cheng Jing, Sen Li, Hao Liu, Xin Wang, Zheng-Yu Jin, Yi-Ning Wang

**Affiliations:** 1 Department of Radiology, State Key Laboratory of Complex Severe and Rare Diseases, Chinese Academy of Medical Sciences and Peking Union Medical College, Peking Union Medical College Hospital, No. 1, Shuaifuyuan, Dongcheng District, Beijing 100730, China; 2 Department of Cardiology, State Key Laboratory of Complex Severe and Rare Diseases, Chinese Academy of Medical Sciences and Peking Union Medical College, Peking Union Medical College Hospital, No. 1, Shuaifuyuan, Dongcheng District, Beijing 100730, China; 3 Department of Research & Development, Yizhun Medical AI Co., Ltd., 12th Floor 12, Block A, Beihang Zhizhen Building, No. 7 Zhichun Road, Haidian District, Beijing 100088, China

**Keywords:** pulmonary arterial hypertension, radiomics, native T1 mapping

## Abstract

(1) Background: Novel markers for predicting the short-term therapeutic effect of pulmonary arterial hypertension (PAH) to assist in the prompt initiation of tailored treatment strategies are greatly needed and highly desirable. The aim of the study was to investigate the role of cardiac magnetic resonance (CMR) native T1 mapping radiomics in predicting the short-term therapeutic effect in PAH patients; (2) Methods: Fifty-five PAH patients who received targeted therapy were retrospectively included. Patients were subdivided into an effective group and an ineffective group by assessing the therapeutic effect after ≥3 months of treatment. All patients underwent CMR examinations prior to the beginning of the therapy. Radiomics features from native T1 mapping images were extracted. A radiomics model was constructed using the support vector machine (SVM) algorithm for predicting the therapeutic effect; (3) Results: The SVM radiomics model revealed favorable performance for predicting the therapeutic effect with areas under the receiver operating characteristic curve of 0.955 in the training cohort and 0.893 in the test cohort, respectively. With the optimal cutoff value, the radiomics model showed accuracies of 0.909 and 0.818 in the training and test cohorts, respectively; (4) Conclusions: The CMR native T1 mapping-based radiomics model holds promise for predicting the therapeutic effect in PAH patients.

## 1. Introduction

Pulmonary arterial hypertension (PAH) is characterized by the obliterative vasculopathy of distal pulmonary circulation leading to severe elevation in pulmonary pressure and pulmonary vascular resistance [1]. The prognosis of PAH is poor, with documented 1-, 3- and 5-year survival rates of 68.0%, 38.9% and 20.8% if left untreated [2]. The treatment goal is to achieve or maintain a low-risk status. During the last several decades, significant improvements in patient prognosis have occurred with the reported 1- and 3-year survival rates reaching 92.1% and 75.1%, respectively [3]. The improvements can be attributed not only to the availability of target therapies but also to the necessary escalation of therapies based on a systematic assessment of the clinical response [4,5,6]. In detail, further treatment strategies rely on the effect seen 3–6 months after the initial therapy according to the 2015 European Society of Cardiology (ESC)/European Respiratory Society (ERS) pulmonary hypertension guidelines. The therapy should be continued if a low-risk status has been achieved or maintained. Escalation to triple combination therapy or maximal medical therapy is recommended if the patient remains or deteriorates to an intermediate- or high-risk status [7]. However, updating treatment strategies after months of therapy carries the potential for deterioration and might affect the long-term prognosis, given that PAH is a progressive disease. Novel markers for predicting the short-term therapeutic effect of PAH to assist in the prompt initiation of tailored treatment strategies are greatly needed and highly desirable.

Cardiac magnetic resonance (CMR) native T1 mapping permits the evaluation of longitudinal relaxation times (T1) within tissue in the absence of contrast agents [8,9]. Native T1 mapping has been found to hold a role in the diagnostic and prognostic evaluation of PAH [10,11]. To date, the value of native T1 mapping for predicting the short-term therapeutic effect of PAH has not been investigated. Radiomics is an emerging field that aims to extract a large number of quantitative features from medical images using data characterization algorithms. It has the potential to uncover disease characteristics that are difficult to identify by the naked human eye or that can not be assessed by conventional measurements [12,13,14]. Moreover, radiomics is independent of dedicated image acquisition and can be applied to existing routinely acquired images [15,16]. Radiomics provides a novel method for quantitative image analysis that might greatly augment diagnostic and predictive capabilities [17]. It has achieved great success in oncology, such as tumor classification, prediction of treatment response and prognostication. Radiomics has gradually been adopted in cardiology in the past few years [13,18]. The aim of this study was to investigate the value of radiomics based on native T1 mapping for predicting the short-term target therapeutic effect in PAH patients, which, to our knowledge, has not yet been evaluated in any published studies.

## 2. Materials and Methods

### 2.1. Patient Population

The institutional review board of Peking Union Medical College Hospital approved this retrospective study and the need for written informed consent was waived. Patients with diagnosed PAH who received targeted therapy at our institution between July 2019 and December 2021 were retrospectively included. The following criteria needed to be met for enrollment: (1) PAH cases had been confirmed by right heart catheterization (RHC) receiving targeted therapy according to baseline risk status; (2) Patients had undergone CMR examination prior to the beginning of the therapy and re-assessment of the risk status to evaluate therapeutic effect ≥3 months after therapy. Meanwhile, the exclusion criteria were as follows: (1). The CMR image quality was insufficient for evaluation; (2) The patient did not receive targeted therapy, or the therapeutic effect was unclear due to the absence of the re-assessed risk status at 3 months after therapy. The risk status was evaluated according to the 2015 ESC/ERS pulmonary hypertension guidelines [7]. Patients who achieved or maintained a low-risk status after therapy were assigned to the effective group (33 patients). Patients who showed intermediate- or high-risk after therapy were assigned to the ineffective group (22 patients). A total of 55 patients were included for analysis. The demographic and clinical characteristics were retrieved by reviewing the electronic medical records.

### 2.2. CMR Examination

CMR images of all patients were acquired by using a 3T scanner (MAGNETOM Skyra, Siemens Healthineers, Erlangen, Germany). An 18-element body matrix coil and a 32-element spine array coil were used for image acquisition. A 4-lead vector cardiogram was used for electrocardiographic gating. Cine images were acquired with an electrocardiographic-gated 2-dimensional balanced steady-state free precession sequence during multiple breath-holds. The key scanning parameters for the acquisition of cine images were as follows: repetition time (TR): 3.3 ms; echo time (TE): 1.43 ms; flip angle (FA): 55–70 degrees; voxel size: 1.6 × 1.6 × 6.0 mm; temporal resolution: 45.6 ms; and bandwidth: 962 Hz/pixel. Moreover, 2-, 3- and 4-chamber long-axis and 9 short-axis slices covering the left ventricle (LV) were acquired. Native T1 images were acquired using a Modified Look-Locker inversion recovery (MOLLI) sequence in identical imaging locations, including a 4-chamber long-axis slice and 3 short-axis slices. Acquisition schemas 5(3)3 and 4(1)3(1)2 were applied before and after the administration of a gadolinium-based contrast medium (gadobenate dimeglumine, Beijing BEILU Pharmaceutical CO LTD., Peking, China, 0.15 mmol/kg), respectively. Other parameters were as follows: TR/TE/FA, 2.7/1.12 ms/20 degrees and voxel size, 1.4 × 1.4 × 8.0 mm. Late gadolinium enhancement (LGE) images were collected by a 2-dimensional phase-sensitive inversion-recovery gradient echo pulse sequence with a breath-hold 15 min after the injection of the contrast material. Parameters of the sequence were as follows: TR/TE/FA, 5.2 ms/1.96 ms/20 degrees and voxel size, 1.4 × 1.4 × 8.0 mm.

### 2.3. CMR Imaging Analysis

**Figure 1:**
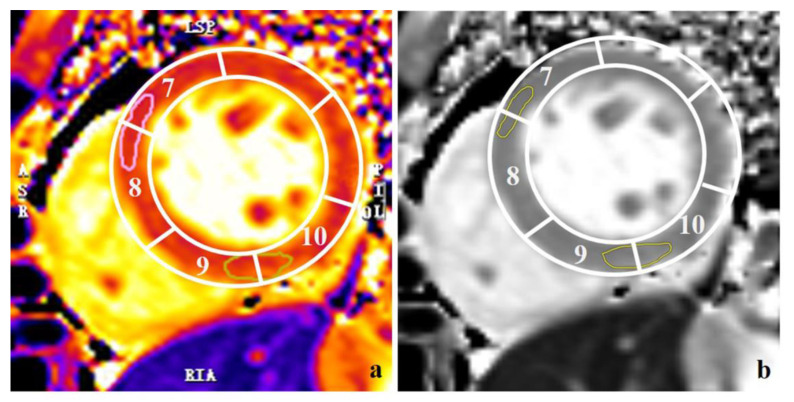
Examples of the measurement of the native T1 values and segmentation of the regions of interest (ROIs) at ARVIP and IRVIP. (**a**). Native T1 values at anterior right ventricle insertion point (ARVIP) and inferior right ventricle insertion point (IRVIP) were measured by delineated regions of interest (ROIs) from the mid-cavity short axis slice between myocardial segments 7/8 and 9/10. The pink circle represents the ROI in ARVIP. The yellow circle represents the ROI in IRVIP. (**b**). Radiomics features were extracted from the segmented ROIs (yellow circles) at ARVIP and IRVIP by using the dedicated platform.

Conventional CMR images of all PAH patients were reviewed and analyzed by a radiologist with 8 years of experience in CMR supervised by a senior radiologist with 15 years of experience in CMR. The presence of LGE was documented. Cardiac function and native T1 values were measured semiautomatically using a dedicated CMR post-processing software CVI42 (version 5.9, Circle Cardiovascular Imaging, Calgary, AB, Canada). Performers were blinded to the subjects’ clinical information when delineating myocardial contours. After the images were uploaded, their brightness was adjusted to ensure optimal endocardial/blood pool discrimination. Conventional functional parameters including LV and right ventricle (RV) stroke volume (SV), end-diastolic volume (EDV), end-systolic volume (ESV) index, and ejection fraction (EF) were measured by manually contouring LV and RV endocardial borders in all short-axial cine images in end-diastolic and end-systolic stages using a point-and-click approach. The values of volume measurements indexed by body surface area were recorded. Native T1 values at the anterior right ventricle insertion point (ARVIP) and the inferior right ventricle insertion point (IRVIP) were measured by delineated regions of interest (ROIs) from the mid-cavity short axis slice between myocardial segments 7/8 and 9/10, respectively (Figure 1a) [19,20].

### 2.4. Radiomics Features Extraction

Image segmentation and radiomics feature extraction were performed using the in-house software “DARWIN intelligent research platform” launched by the Yizhun Medical AI technology limited company (https://www.yizhun-ai.com, accessed on 16 June 2022). Digital Imaging and Communications in Medicine format T1 mapping images created by the Siemens 3.0 T magnetic resonance imaging scanner were exported and uploaded to the platform for radiomics analysis. The ROIs of all patients were delineated by the junior radiologist (8 years of experience in CMR evaluation) manually both at the ARVIP and IRVIP from the mid-cavity short axis slice (Figure 1b). The ROIs were delineated by the senior radiologist (15 years of experience in CMR evaluation) in 30 randomly selected patients (20 cases in the effective group and 10 cases in the ineffective group) for evaluation of the inter-observer variability. Complex pre-processing steps such as normalization or inhomogeneity correction were unnecessary for T1 mapping images before radiomics analysis given that each pixel in the map represents the objective corresponding T1 values of the tissues under the same scanning conditions [21,22]. The software enables the automatic extraction of radiomics features from the segmented ROIs. The calculation of features was based on the publicly available library PyRadiomics (https://pyradiomics.readthedocs.io/en/latest/features.html, accessed on 16 June 2022). Shape and size features were obtained from the original images. Four levels of wavelet decompositions, Gradient, Local Binary Patterns, Laplacian of Gaussian, Square, Square Root, Logarithm, and Exponential filters were used to transform the original images to 11 types of derived images for the extraction of more features. First-order statistics features and texture-based features including gray level co-occurrence matrix (GLCM) features, gray level dependence matrix (GLDM) features, gray level run length matrix (GLRLM) features, gray level size zone matrix (GLSZM) features and neighboring gray tone difference matrix (NGTDM) features were obtained from both the original and derived images. 

### 2.5. Radiomics Model Construction

**Figure 2:**
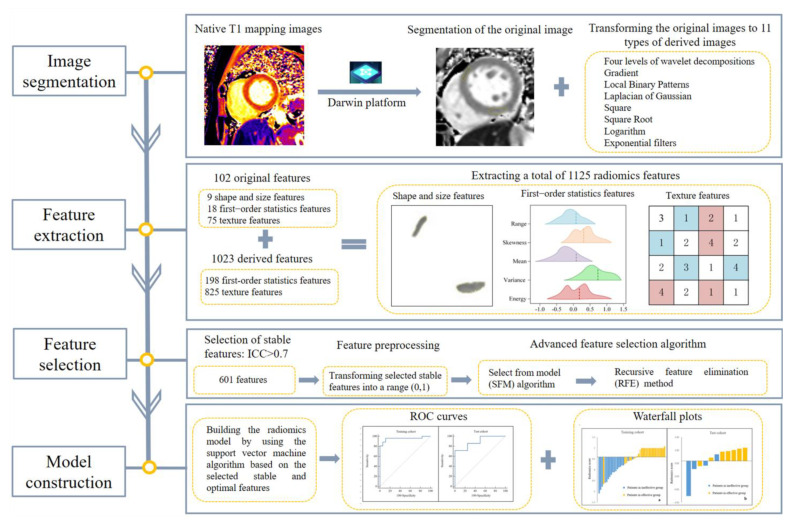
Workflow of the radiomics model construction.

Feature selection and radiomics model construction were processed using in-house software programmed with Python Scikit-learn package (Python version 3.7, Scikit-learn version 0.21, http://scikit-learn.org/, accessed on 16 June 2022). The patients were split randomly into training and test datasets with a ratio of 8:2 using a stratified sampling strategy. Subsequently, 44 patients (26 cases in the effective group and 18 cases in the ineffective group) were allocated to the training cohort and 11 patients (7 cases in the effective group and 4 cases in the ineffective group) were allocated to the test cohort. Only features with an interclass correlation coefficient (ICC) > 0.7 were selected for further analysis. Feature pre-processing was performed by scaling and transforming all selected features into a range (0,1) using the standardization method. The select from model (SFM) algorithm was attached to the standardized features for selecting important features based on their importance weights. Another feature selection algorithm recursive feature elimination (RFE) was employed in addition after performing the SFM method. The RFE algorithm enables the identification of the most relevant features and the removal of the weakest features. Following the selection of stable and optimal radiomics features through the above-mentioned composite feature selection methods, a support vector machine (SVM) model using the grid search approach was built. Five-fold cross validation was performed to facilitate the model performance. The radiomics scores of each patient were recorded. The workflow of the development of the radiomics model was provided in Figure 2.

### 2.6. Statistical Analysis

SPSS (version 24; IBM Corporation, Armonk, NY, USA) was used to perform statistical analyses. The Kolmogorov-Smirnov test was used to test for normality for continuous variables. The variables were shown as the mean ± SD (standard deviation) values if normally distributed and compared using a Student *t*-test. Discrete variables were presented as frequencies, and the chi-square test was used to test for differences. The interobserver variability of the radiomics features was assessed with ICC. The receiver operating characteristic (ROC) curves and the area under curve (AUC) were calculated. The Sensitivity, specificity, positive predictive value (PPV) and negative predictive value (NPV) were also calculated.

## 3. Results

### 3.1. Clinical and Conventional CMR Characteristics

**Figure 3:**
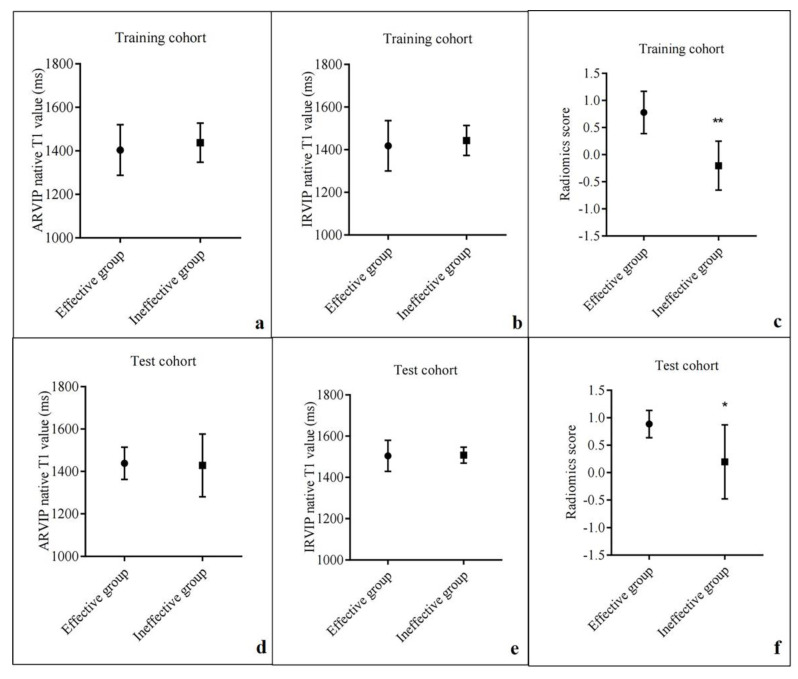
Mean values with error bars for ARVIP, IRVIP and radiomics scores in training and test cohorts. The centers represent the mean values, and the bars denote standard value (SD). ** *p* < 0.001; * *p* < 0.05. (**a**–**c**). Comparison of the native ARVIP T1 value (**a**), native IRVIP T1 value (**b**) and radiomics score (**c**) between effective group and ineffective group in training cohort. (**d**–**f**). Comparison of the native ARVIP T1 value (**d**), native IRVIP T1 value (**e**) and radiomics score (**f**) between effective group and ineffective group in test cohort.

The 55 patients included 44 idiopathic pulmonary arterial hypertension patients, 4 heritable pulmonary arterial hypertension patients, 6 connective tissues disease-related pulmonary arterial hypertension patients and 1 patient with congenital heart disease. The comparisons of the clinical and conventional CMR parameters of patients in the effective and ineffective groups were summarized in Table 1. Overall, 60.60% (20 cases) of the patients in the effective group and 22.74% (5 cases) of the patients in the ineffective group showed a low-risk status before therapy (*p* = 0.022). None of the CMR-based cardiac functional parameters significantly differed between patients in the effective and ineffective groups. Two patients did not undergo contrast-enhanced examinations. The image quality of LGE images was insufficient in one case. The remaining 53 patients showed LGE in both ARVIP and IRVIP. Moreover, 37 patients showed additional interventricular septum LGE. The analyses of the demographical and clinical characteristics in training and test cohorts are presented in Table 2. There were no significant differences in terms of most of the clinical and CMR-based cardiac functional parameters between the training and test cohorts. Native T1 values between patients in the effective and the ineffective groups were not significantly different in either the training (ARVIP: 1403.92 ± 116.30 ms vs. 1437.61 ± 89.97 ms, *p* = 0.308; IRVIP: 1418.31 ± 118.28 ms vs. 1443.28 ± 70.32 ms, *p* = 0.427) or the test (ARVIP: 1438.14 ± 76.00 ms vs. 1428.25 ± 148.02 ms, *p* = 0.884; IRVIP: 1504.43 ± 75.45 ms vs. 1507.75 ± 38.68 ms, *p* = 0.937) cohort (Figure 3a,b,d,e).

### 3.2. Radiomics Model Construction and Evaluation

**Figure 4:**
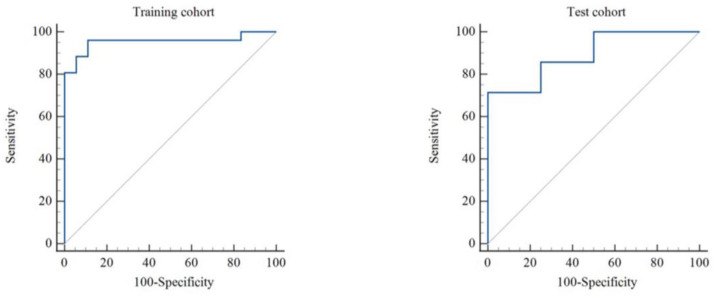
ROC curves of radiomics model in the training and test cohorts.

**Figure 5:**
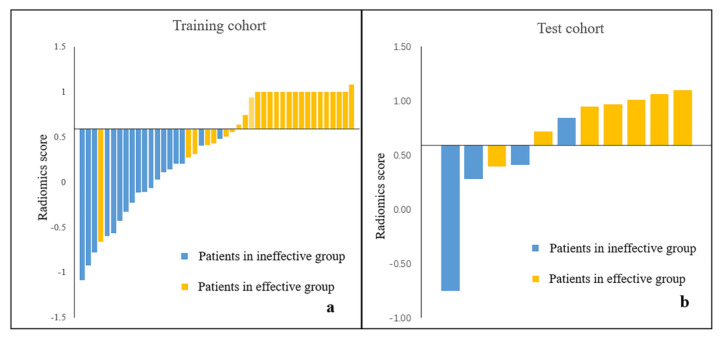
Waterfall plots of the radiomics scores of PAH patients. (**a**) Radiomics scores in PAH patients with different therapeutic effect in training cohort. (**b**) Radiomics scores in PAH patients with different therapeutic effect in test cohort.

Altogether 1125 radiomics features were obtained from the segmented ROI of each patient, of which 102 features (9 shape and size features, 18 first-order statistics features and 75 texture features) were extracted from the original images and 1023 features (198 first-order statistics features and 825 texture features) were extracted from the images processed with the variable filters. A total of 601 features with ICC values ≥ 0.7 were selected for further feature selection and SVM model construction, including 5 shape and size features and 143 first-order, 169 GLCM, 80 GLDM, 102 GLRLM, 77 GLSZM and 25 NGTDM features. The radiomics scores in the effective group were significantly higher than those in the ineffective group in both the training cohort (0.778 ± 0.39 vs. −0.203 ± 0.451, *p* < 0.001) and the test cohort (0.885 ± 0.248 vs. 0.196 ± 0.676, *p* = 0.034) (Figure 3c,f). The radiomics model revealed an AUC of 0.955 and 0.893 in the training and test cohorts, respectively (Figure 4). With the cutoff value of 0.589, the radiomics model showed accuracies of 0.909 in the training cohort and 0.818 in the test cohort for predicting the short-term therapeutic effect in PAH patients (Figure 4). Detailed model performances were shown in Table 3. Waterfall plots for the distribution of the radiomics score and the therapeutic effect in individuals in the training and test cohorts were presented in Figure 5.

## 4. Discussion

The present study developed a native T1-based radiomics model for predicting the therapeutic effect in PAH patients. The major finding was that the SVM radiomics model from native T1 mapping images holds the potential to predict the short-term therapeutic effect in PAH patients.

Native T1 values were increased in PAH patients, especially at ARVIP and IRVIP T1 values, which was considered to be caused by the exaggeration of the myocardial disarray and plexiform fibrosis wherein fibers from the RV and LV cross due to the combination of RV hypertrophy and increased shear forces up the interventricular septum of PAH patients [10,23]. The study carried out by Reiter et al. [11] also showed that ARVIP and IRVIP native T1 times were longer in patients with pulmonary hypertension and significantly correlated with the LV eccentricity index (r = 0.72). The meta-analysis conducted by Alabed et al. [10] reported that native T1 values in PAH patients are on average 9% higher than those of healthy controls, and that ARVIP/IRVIP native T1 was associated with poor RV function and dilation in PAH patients. Roller et al. [19] found that ARVIP/IRVIP native T1 values seem to be indicative of reverse myocardial tissue remodeling after balloon pulmonary angioplasty. Whereas, neither ARVIP nor IRVIP native T1 values significantly differed between the effective group and the ineffective group in either the training or the test cohort in the current study. This result may partly be due to the fact that much of the information available within T1 mapping images was not optimally utilized. 

The innovative radiomics method allows for extracting a lot of information hidden in imaging and utilizing machine learning algorithms to build models to assist in the prediction of important clinical outcomes. Prior studies reported that radiomics might facilitate the prediction of the treatment response in several disease entities, such as hepatocellular carcinoma, pancreatic cancer, rectal cancer and gastric cancer [24,25,26,27]. As for the application of radiomics in cardiology, the study conducted by Son et al. [22] revealed that a native T1 radiomics model could differentiate thrombi from tumors better than the mean T1 value (AUC 0.98 vs. 0.86). Ma et al. [28] reported that native T1 mapping-based radiomics showed superior performance for the diagnosis of microvascular obstruction compared to T1 values. Neisius et al. [29] identified that the radiomics analysis of native T1 could discriminate between hypertensive heart disease and hypertrophic cardiomyopathy and provided incremental value over global native T1 mapping. Another study indicated that the radiomics model derived from native T1 mapping was useful for the prediction of the major adverse cardiac events in patients with acute ST-segment elevation myocardial infarction. The present study for the first time built a native T1 mapping-based radiomics model for predicting the short-term therapeutic effect of PAH patients. Similar to the published literature, the radiomics model in the present study showed favorable performance for predicting the therapeutic effect in PAH patients with AUCs of 0.955 and 0.893 in the training and test cohorts, respectively. Meanwhile, the accuracies were 0.909 in the training cohort and 0.818 in the test cohort.

This study has several limitations. Firstly, the small sample size was a major limitation of this retrospective analysis. Future studies with inclusion of more patients should be conducted for the further improvement of the model performance as it is widely acknowledged that the quality of radiomics models is highly dependent on the size of the training dataset. Secondly, this was a single-center study and the performance of the existing radiomics model lacks external validation. As such, the generalizability of the findings could not be commented on. Further studies that include multi-center cohorts for external validation are warranted. Finally, this study merely utilized machine learning algorithms to build the model; the state-of-the-art deep-learning techniques merit further investigation.

## 5. Conclusions

In a word, this study explored the possibility of using a radiomics model based on native T1 mapping for the prediction of therapeutic effects in PAH patients. The preliminary results suggest that the radiomics model built on native T1 mapping has the potential to predict the short-term therapeutic effects in PAH patients, which might supplement the adjuvant evaluation of PAH patients and facilitate the timely selection of tailored treatment strategies to improve prognosis.

## Figures and Tables

**Table 1:** Demographic and clinical characteristics of PAH patients.

Parameter	Effective Group, *n* = 33	Ineffective Group, *n* = 22	*p*
Age, yrs	33 ± 17	32 ± 12	0.977
Gender			0.157
Females (%)	29 (87.88)	16 (72.73)	
Males (%)	4 (12.12)	6 (27.27)	
Baseline risk status			0.022
Low-risk (%)	20 (60.60)	5 (22.73)	
Intermediate-risk (%)	10 (30.30)	13 (59.09)	
High-risk (%)	3 (9.10)	4 (18.18)	
RHC parameters			
Mean pulmonary arterial pressure (mmHg)	43.36 ± 12.19	52.86 ± 16.82	0.018
Pulmonary arterial wedge pressure (mmHg)	7.88 ± 2.46	11.00 ± 10.29	0.100
Pulmonary vessel resistance (WU)	7.86 ± 4.56	11.05 ± 5.98	0.031
Cardiac functional parameters			
LVEF (%)	61.30 ± 8.45	62.96 ± 11.81	0.545
LV SV index (mL/m^2^)	43.39 ± 17.27	37.49 ± 13.02	0.179
LV EDV index (mL/m^2^)	63.49 ± 15.04	58.97 ± 13.34	0.261
LV ESV index (mL/m^2^)	43.39 ± 17.27	37.49 ± 13.01	0.527
RVEF (%)	42.41 ± 9.77	38.53 ± 12.26	0.199
RV SV index (mL/m^2^)	48.60 ± 19.13	48.57 ± 18.96	0.996
RV EDV index (mL/m^2^)	113.41 ± 34.38	118.95 ± 28.59	0.541
RV ESV index (mL/m^2^)	67.16 ± 28.88	82.81 ± 49.60	0.145
Native T1 time ARVIP (ms)	1411.18 ± 108.86	1435.98 ± 98.46	0.395
Native T1 time IRVIP (ms)	1436.58 ± 115.21	1455.00 ± 69.74	0.505

Data in parentheses are percentages. ARVIP: anterior right ventricle insertion point; IRVIP: inferior right ventricle insertion point; LVEF: left ventricle ejection fraction; LVEDV: left ventricle end-diastolic volume; LVESV: left ventricle end-systolic volume; LV SV: left ventricle stroke volume; PAH: pulmonary arterial hypertension; RHC: right heart catheterization; RVEF: right ventricle ejection fraction; RV EDV: right ventricle end-diastolic volume; RV ESV: right ventricle end-systolic volume; RV SV: right ventricle stroke volume.

**Table 2:** Comparison of the clinical and conventional CMR characteristics in training and test cohort.

Parameter	Training Cohort, *n* = 44	Test Cohort, *n* = 11	*p*
Age, yrs	33 ± 16	32 ± 10	0.897
Gender			0.387
Females (%)	35 (79.55)	10 (90.91)	
Males (%)	9 (20.45)	1 (9.09)	
Baseline risk status			0.530
Low-risk (%)	19 (43.18)	6 (54.55)	
Intermediate-risk (%)	20 (45.45)	3 (27.27)	
High-risk (%)	5 (11.37)	2 (18.18)	
RHC parameters			
Mean pulmonary arterial pressure (mmHg)	48.74 ± 15.45	40.91 ± 10.15	0.118
Pulmonary arterial wedge pressure(mmHg)	9.41 ± 7.43	7.82 ± 2.094	0.488
Pulmonary vessel resistance (WU)	9.90 ± 5.58	6.25 ± 3.23	0.009
Cardiac functional parameters			
LVEF (%)	62.61 ± 9.35	59.38 ± 11.85	0.336
LV SV index (mL/m^2^)	41.64 ± 16.76	38.59 ± 11.872	0.573
LV EDV index (mL/m^2^)	62.68 ± 15.37	57.69 ± 9.34	0.309
LV ESV index (mL/m^2^)	24.28 ± 8.32	23.28 ± 7.44	0.719
RVEF (%)	40.78 ± 11.13	41.16 ± 10.43	0.920
RV SV index (mL/m^2^)	49.69 ± 20.20	44.20 ± 12.07	0.393
RV EDV index (mL/m^2^)	120.08 ± 32.08	95.69 ± 24.65	0.029
RV ESV index (mL/m^2^)	72.56 ± 28.77	76.86 ± 67.70	0.746
Native T1 time ARVIP (ms)	1417.70 ± 106.51	1434.55 ± 100.32	0.629
Native T1 time IRVIP (ms)	1428.52 ± 101.21	1505.64 ± 62.19	0.020

Data in parentheses are percentages. ARVIP: anterior right ventricle insertion point; IRVIP: inferior right ventricle insertion point; LVEF: left ventricle ejection fraction; LVEDV: left ventricle end-diastolic volume; LVESV: left ventricle end-systolic volume; LV SV: left ventricle stroke volume; RHC: right heart catheterization; RVEF: right ventricle ejection fraction; RV EDV: right ventricle end-diastolic volume; RV ESV: right ventricle end-systolic volume; RV SV: right ventricle stroke volume.

**Table 3:** Performances of radiomics models for prediction of the short-term therapeutic effect in PAH patients.

	Cut-Off Value	AUC	Accuracy	Specificity	Sensitivity	PPV	NPV
Training cohort	0.589	0.955	0.909	0.889	0.962	0.892	0.9375
Test cohort	0.589	0.893	0.818	1.000	0.714	1.000	0.667

AUC: area under curve; PAH: pulmonary arterial hypertension.

## Data Availability

The data presented in this study are available on request from the corresponding author.
